# Altered infective competence of the human gut microbiome in COVID-19

**DOI:** 10.1186/s40168-023-01472-7

**Published:** 2023-03-09

**Authors:** Laura de Nies, Valentina Galata, Camille Martin-Gallausiaux, Milena Despotovic, Susheel Bhanu Busi, Chantal J. Snoeck, Lea Delacour, Deepthi Poornima Budagavi, Cédric Christian Laczny, Janine Habier, Paula-Cristina Lupu, Rashi Halder, Joëlle V. Fritz, Taina Marques, Estelle Sandt, Marc Paul O’Sullivan, Soumyabrata Ghosh, Venkata Satagopam, CON-VINCE Consortium, Geeta Acharya, Gloria Aguayo, Wim Ammerlaan, Ariane Assele-Kama, Christelle Bahlawane, Katy Beaumont, Nadia Beaupain, Lucrèce Beckers, Camille Bellora, Fay Betsou, Sandie Boly, Dirk Brenner, Eleftheria Charalambous, Emilie Charpentier, Manuel Counson, Brian De Witt, Olivia Domingues, Claire Dording, Bianca Dragomir, Tessy Fautsch, Jean-Yves Ferrand, Ana Festas Lopes, Joëlle Véronique Fritz, Manon Gantenbein, Laura Georges, Jérôme Graas, Gael Hamot, Anne-Marie Hanff, Maxime Hansen, Lisa Hefele, Estelle Henry, Margaux Henry, Eve Herkenne, Christiane Hilger, Judith Hübschen, Laetitia Huiart, Alexander Hundt, Gilles Iserentant, Stéphanie Kler, Pauline Lambert, Sabine Lehmann, Morgane Lemaire, Andrew Lumley, Monica Marchese, Sophie Mériaux, Maura Minelli, Alessandra Mousel, Maeva Munsch, Mareike Neumann, Magali Perquin, Achilleas Pexaras, Jean-Marc Plesseria, Lucie Remark, Bruno Santos, Aurélie Sausy, Margaux Schmitt, Sneeha Seal, Jean-Yves Servais, Florian Simon, Chantal Snoeck, Kate Sokolowska, Hermann Thien, Johanna Trouet, Jonathan Turner, Michel Vaillant, Daniela Valoura Esteves, Charlène Verschueren, Tania Zamboni, Pinar Alper, Piotr Gawron, Enrico Glaab, Clarissa Gomes, Borja Gomez Ramos, Vyron Gorgogietas, Valentin Groues, Wei Gu, Laurent Heirendt, Ahmed Hemedan, Sascha Herzinger, Anne Kaysen, Jacek Jaroslaw Lebioda, Tainà Marques, François Massart, Christiane Olesky, Venkata P. Satagopam, Claire Pauly, Laure Pauly, Lukas Pavelka, Guilherme Ramos Meyers, Armin Rauschenberger, Basile Rommes, Kirsten Rump, Reinhard Schneider, Valerie Schröder, Amna Skrozic, Lara Stute, Noua Toukourou, Christophe Trefois, Carlos Vega Moreno, Maharshi Vyas, Xinhui Wang, Anja Leist, Annika Lutz, Claus Vögele, Linda Hansen, João Manuel Loureiro, Beatrice Nicolai, Alexandra Schweicher, Femke Wauters, Tamir Abdelrahman, Estelle Coibion, Guillaume Fournier, Marie Leick, Friedrich Mühlschlegel, Marie France Pirard, Nguyen Trung, Philipp Jägi, Henry-Michel Cauchie, Delphine Collart, Leslie Ogorzaly, Christian Penny, Cécile Walczak, Rejko Krüger, Guy Fagherazzi, Markus Ollert, Feng Q. Hefeng, Patrick May, Paul Wilmes

**Affiliations:** 1 Systems Ecology Group, Luxembourg Centre for Systems Biomedicine, University of Luxembourg, Esch-sur-Alzette, Luxembourg; 2 Clinical and Applied Virology, Department of Infection and Immunity, Luxembourg Institute of Health, Esch-sur-Alzette, Luxembourg; 3 Luxembourg Centre for Systems Biomedicine, LCSB Operations, University of Luxembourg, Esch-sur-Alzette, Luxembourg; 4 Scientific Central Services, Luxembourg Centre for Systems Biomedicine, University of Luxembourg, Esch-sur-Alzette, Luxembourg; 5 Transversal Translation Medicine, Luxembourg Institute of Health, Strassen, Luxembourg; 6 Translational Neuroscience Group, Luxembourg Centre for Systems Biomedicine, University of Luxembourg, Esch-sur-Alzette, Luxembourg; 7 Translational Medicine Operations Hub, Luxembourg Institute of Health, Strassen, Luxembourg; 8 Bioinformatics Core, Luxembourg Centre for Systems Biomedicine, University of Luxembourg, Esch-sur-Alzette, Luxembourg; 9 Deep Digital Phenotyping Research Unit, Department of Precision Health, Luxembourg Institute of Health, Strassen, Luxembourg; 10 Department of Infection and Immunity, Luxembourg Institute of Health, Esch-Sur-Alzette, Luxembourg; 11 Department of Dermatology and Allergy Centre, Odense University Hospital, Odense, Denmark; 12 Department of Life Sciences and Medicine, Faculty of Science, Technology and Medicine, University of Luxembourg, 6, Avenue du Swing, L-4367 Belvaux, Luxembourg

**Keywords:** COVID-19, Metagenomics, Metatranscriptomics, Gut microbiome, SARS-CoV-2

## Abstract

**Background:**

Infections with SARS-CoV-2 have a pronounced impact on the gastrointestinal tract and its resident microbiome. Clear differences between severe cases of infection and healthy individuals have been reported, including the loss of commensal taxa. We aimed to understand if microbiome alterations including functional shifts are unique to severe cases or a common effect of COVID-19. We used high-resolution systematic multi-omic analyses to profile the gut microbiome in asymptomatic-to-moderate COVID-19 individuals compared to a control group.

**Results:**

We found a striking increase in the overall abundance and expression of both virulence factors and antimicrobial resistance genes in COVID-19. Importantly, these genes are encoded and expressed by commensal taxa from families such as Acidaminococcaceae and Erysipelatoclostridiaceae, which we found to be enriched in COVID-19-positive individuals. We also found an enrichment in the expression of a betaherpesvirus and rotavirus C genes in COVID-19-positive individuals compared to healthy controls.

**Conclusions:**

Our analyses identified an altered and increased infective competence of the gut microbiome in COVID-19 patients.

Video Abstract

**Supplementary Information:**

The online version contains supplementary material available at 10.1186/s40168-023-01472-7.

## Background

Coronavirus disease 2019 (COVID-19), which is caused by the severe acute respiratory syndrome coronavirus 2 (SARS-CoV-2), was declared a global pandemic by the World Health Organization (WHO). COVID-19 exhibits a high degree of clinical heterogeneity, ranging from asymptomatic to severe disease, and may be accompanied by a poor outcome and a relatively high mortality rate [1]. As of 17 October 2022, more than 621 million confirmed SARS-CoV-2 infections and 6.5 million COVID-19-related deaths have been reported [2]. Although COVID-19 is primarily considered a respiratory disease, it clinically often presents with general (fever, myalgia, and/or fatigue) and respiratory symptoms (cough and/or dyspnea). Moreover, an emergence of new variants has led to the more frequent presentation of gastrointestinal symptoms (appetite loss, nausea, vomiting, and diarrhea) [3], indicating a potential involvement of the gastrointestinal tract in COVID-19. More specifically, SARS-CoV-2 has been shown to be able to infect and replicate in enterocytes in vitro [4]. In fact, viral RNA can be detected in fecal samples even after resolution of respiratory symptoms [5]. Additionally, SARS-CoV-2 infections are associated with alterations to the gut microbiome composition that persist for at least 6 months after the initial infection [6]. Thus, an imbalance in the gut microbiome can be linked to disease severity and increased concentrations of inflammatory markers, as well as an increased post-COVID-19 risk, understood as a wide range of symptoms persisting four or more weeks after the initial SARS-CoV-2 infection [6, 7].

Stable ecosystems are important for colonization resistance to pathogens [8]. As such, host and SARS-CoV-2-mediated immune dysregulation and dysbiosis may predispose patients to co-infections or secondary infections of the respiratory and gastrointestinal tracts. In addition, co-infecting microorganisms may alter the intensity of the host immune response [9], thus significantly influencing severity and outcome of the disease. For instance, co-infections with viruses (rhinovirus/enterovirus, respiratory syncytial virus, influenza virus, non–SARS-CoV-2 Coronavirus) [10], bacteria (*Mycoplasma pneumoniae*, *Pseudomonas aeruginosa*, *Haemophilus influenzae*, *Klebsiella pneumoniae, Streptococcus pneumoniae, Staphylococcus aureus*) [11, 12], or fungi (*Candida spp., Aspergillus spp.*) [13] have been described among SARS-CoV-2-positive cases in different study set-ups. In particular, bacterial co-infections in hospitalized and intensive care unit patients with COVID-19 are associated with prolonged ventilation and an increased mortality rate [11, 14]. Furthermore, hospital-acquired infections with multi-drug-resistant (MDR) pathogens are also linked with increased mortality in COVID-19 patients [15]. These reports collectively suggest a clear shift in COVID-19 patients with respect to an increased abundance of pathogens and potential for harm. Moreover, these shifts may further manifest themselves in relation to the *infective competence*, i.e., the propensity for virulence and increased antibiotic resistance, in the gut microbiome as a consequence of an increased capacity to cause infections.

Major factors that contribute to the success of some of the pathogens highlighted above are virulence factors (VFs). Virulence factors including cell-surface structures, adhesins, siderophores, endo-, and exotoxins enable pathogens to undergo quick adaptive shifts, invade and colonize host niches, as well as evade innate and adaptive immune mechanisms of the host, resulting in inflammation and clinical manifestations of the disease. Another factor facilitating colonization of pathogens, through prevention of effective treatment, is antimicrobial resistance (AMR). Even though AMR is an ancient and natural phenomenon [16], it is usually linked to the human influence on the environment and the use of antibiotics. Overuse of antibiotics is hypothesized to also contribute to the broader problem of antimicrobial resistance [17]. Moreover, although not a VF by itself, AMR shares common characteristics with VFs [18]. Specifically, AMR and VFs: (1) are necessary for the survival of pathogens under unfavourable conditions [19]; (2) can be transmitted between species by horizontal gene transfer [20]; and (3) both processes make use of similar systems, e.g., cell wall alterations, efflux pumps, porins, and two-component systems to activate or repress expression of various genes [18, 21]. Thus, in response to host defence mechanisms and environmental challenges, communities of microorganisms, i.e., microbiomes, may alter their “*infective competence”.* The *infective competence* is defined as the ability of microorganisms to constantly adapt and evolve, utilizing VFs and AMR mechanisms, resulting in increased survival, invasion, or growth. Importantly, the combination of host-driven factors, i.e., immune system-mediated effects and antimicrobial peptides, and unfavourable gastrointestinal conditions, e.g., low pH, disruption of the mucus layer, niche competition with other taxa, may confer transiently a selective advantage to a pathogenic lifestyle [22, 23]. This may be reflected in the entire gut microbiome, possibly altering the *infective competence* of the endogenous taxa and subsequently giving rise to pathobiont-dominated communities.

Here, we addressed questions pertaining to the effect of SARS-CoV-2 infection on the endogenous gut microbiome in COVID-19 cases compared to healthy controls using systematic, high-resolution multi-omic data, including metagenomics and metatranscriptomics with a particular focus on VFs and antimicrobial resistance genes (ARGs). We find that mild, i.e. asymptomatic-to-moderate, COVID-19 does not alter the overall composition of the gut microbiome, unlike the drastic microbiota changes reported previously in severe cases. Importantly, we find that a mild progression of COVID-19 affects the *infective competence* of gut microbiota, wherein taxa encode and express genes facilitating their survival and/or growth. We find specific families such as Acidaminococcaceae and Erysipelatoclostridiaceae to be encoding for and expressing VFs and ARGs, significantly more in individuals with COVID-19. Collectively our data also demonstrates a significantly higher *infective competence* of the endogenous microbiome, suggesting that infection with SARS-CoV-2 may mediate co-infections in the longer term.

## Results

### Taxonomic and functional profiles indicate minimal changes in COVID-19

**Fig. 1:**
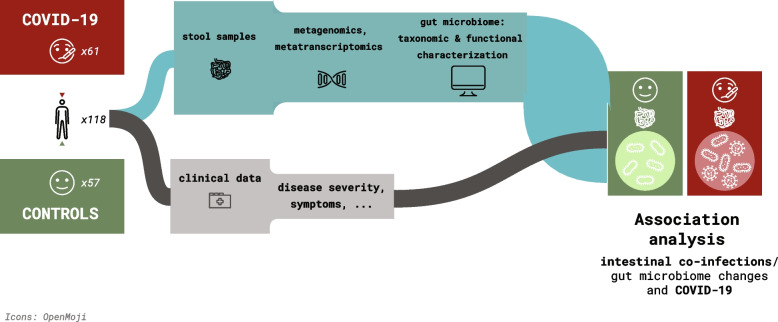
Sample collection and study design. Schematic of the project study design, including cohort composition, and data analyses

**Fig. 2:**
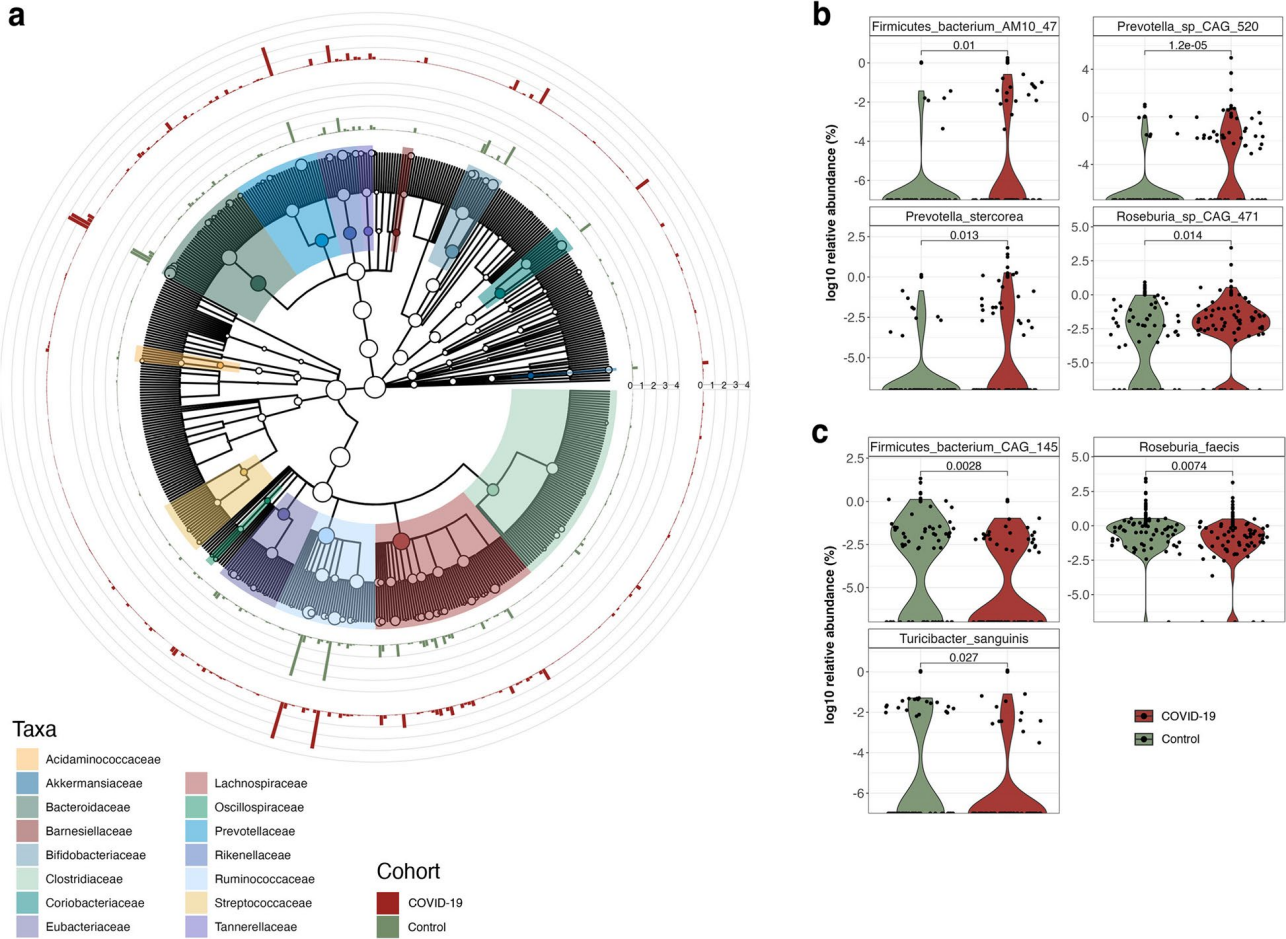
Composition of the microbial community. **a** Cladogram representing the microbial community profiles in COVID-19 patients (red) and control group (green). The outer rings represent the relative abundance (%) of the microbial community. **b** Relative abundance of bacterial species significantly enriched in COVID-19 patients compared to the control group [*adj.p* < 0.05; Wilcoxon rank-sum test]. **c** Relative abundance of bacterial species significantly decreased in COVID-19 patients compared to the control group [*adj. p* < 0.05; Wilcoxon rank-sum test]

COVID-19 studies have reported an altered gut microbiota composition of hospitalized and critical COVID-19 patients. However, limited attention has been paid to milder forms of COVID-19. Thus, we assessed whether gut microbiota composition was altered in COVID-19 individuals compared to healthy controls. Overall, the gut microbiome compositions, based on the alpha- and beta-diversity metrics, of 61 COVID-19 and 57 individuals from the control group were similar (Fig. 1 and Supplementary Figure S1), with an increased abundance of species belonging to the Lachnospiraceae, Ruminococcaceae, Bacteroidaceae, and Bifidobacteriaceae families in COVID-19 (Fig. 2a). We found specific taxonomic differences within the metagenomes, such as an increase in the abundance of AM10 47 (Firmicutes phylum), *Prevotella sp.* CAG 520, *Prevotella stercorea* and *Roseburia sp.* CAG 471 in the COVID-19 group (Fig. 2b), along with a decrease in CAG 145 (Firmicutes phylum), *Roseburia faecis* and *Turicibacter sanguinis* (Fig. 2c). Despite these taxonomic differences, we did not observe any significant changes in the overall functional profile of the microbiome between the COVID-19 and control groups. Along similar lines, we did not find a significant correlation between covariates such as age, sex, COVID-19 severity, and other variables in the COVID-19 and control groups in relation to the taxonomic and functional features.

In light of reports, indicating the potential co-infections with viruses along with SARS-CoV-2, we also assessed the virome (Methods) within the COVID-19 patient and the control groups. We did not observe large differences between the groups. However, we found that genes associated with a specific betaherpesvirus and rotavirus were enriched (*adj. p* < 0.05; one-way ANOVA) in the COVID-19 group (Supplementary Table S2).

### SARS-CoV-2 is associated with increased abundance and expression of virulence factors

**Fig. 3:**
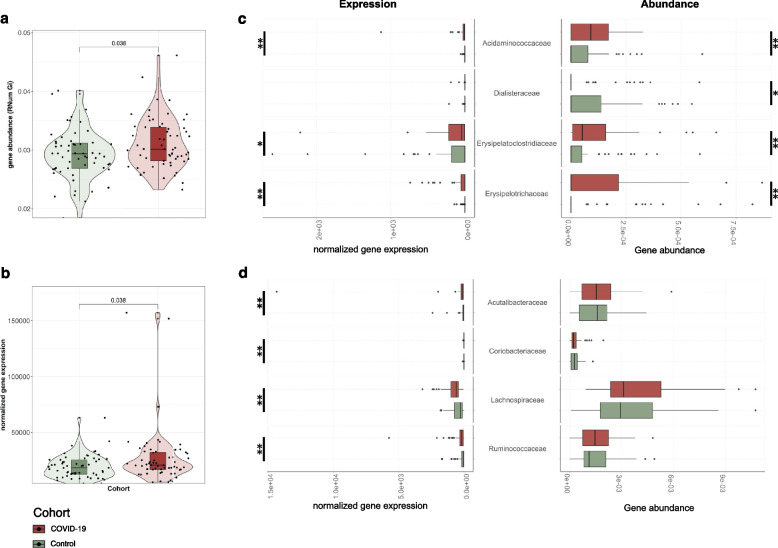
Abundance of virulence factors in the microbial community. **a** Overall abundance (metagenome) of virulence factors encoded by the microbiome of COVID-19 patients and control group. The significance of the differential abundance is indicated with the adjusted *p* value [*adj.p* < 0.05; Wilcoxon rank-sum test]. **b** Overall expression levels (metatranscriptomics) of virulence factors encoded by the microbiome in COVID-19 patients and the control group [*adj.p* < 0.05; Wilcoxon rank-sum test]. **c** Abundance and expression levels of MAG families where a significant increase in encoded and expressed virulence factors was observed in COVID-19 patients [*adj.p* < 0.05; Wilcoxon rank-sum test, * < 0.05, ** < 0.01, *** < 0.001]. **d** Abundance and expression levels of virulence factors in MAGs depicting taxonomic families only demonstrating an increased expression of virulence factors, with no significant difference observed at a metagenomic level [*adj.p* < 0.05; Wilcoxon rank-sum test, * < 0.05, ** < 0.01, *** < 0.001]

SARS-CoV-2 infections have been suggested to predispose patients to co-infections or secondary infections of the respiratory and gastrointestinal tracts. Virulence factors in particular enable (pathogenic) microorganisms to colonize host niches and establish infections. We used PathoFact [24] to assess the prevalence of VFs in the co-assembled metagenomic and metatranscripomic data. PathoFact was designed to contextualize the genomic data and classify VFs and ARGs, allowing to assess the *infective competence* of taxa. To obtain a comprehensive overview of actual gene expression, we complemented metagenomic analyses with metatranscriptomic information conferring information regarding the transcription levels of identified VFs. Based on the metagenomic data, we found a significant increase (*adj. p* < 0.05; Wilcoxon rank-sum test) in alpha diversity (Supplementary Figure S2) as well as the overall abundance of VFs in the COVID-19 group compared to the control group (Fig. 3a). The metatranscriptomic information further confirmed that these VFs demonstrated significantly increased expression levels (*adj. p* < 0.05; Wilcoxon rank-sum test) in the COVID-19 group compared to the control group (Fig. 3b).

To link the prevalence and expression of the identified VFs to the taxa within the microbial community, we reconstructed metagenome-assembled genomes (MAGs) and further leveraged the iterative workflow of the integrated meta-omic pipeline (IMP) [25]. Overall, we found a significant increase in encoded and expressed VFs between the COVID-19 and control groups (*adj. p* < 0.05; Wilcoxon rank-sum test). Our analyses further linked families such as Acidaminococcaceae, Erysipelatoclostridiaceae, and Erysipelotrichaceae with increased expression of VFs in the COVID-19 group (Fig. 3c). Interestingly, the control group exhibited higher gene abundances and expression of VFs only in the Dialisteraceae family. Furthermore, we found that some families (Acutalibacteraceae*,* Coriobacteriaceae*,* Lachnospiraceae, and Ruminococcaceae) demonstrated an increased expression of VFs in the COVID-19 group (Fig. 3d; *adj. p* < 0.05; Wilcoxon rank-sum test), although their respective gene abundances were not different from those found in the control group.

### Expression of antimicrobial resistance increases together with virulence factors

**Fig. 4:**
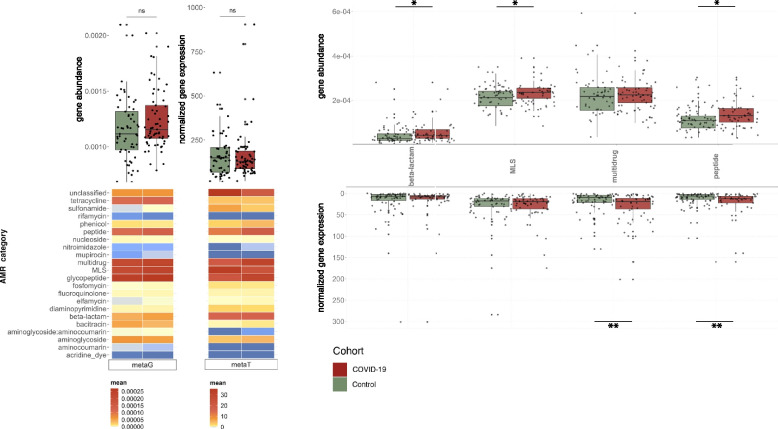
Abundance levels of antimicrobial resistance genes. **a** Overall ARG abundance and expression levels for COVID-19 and control groups (boxplot), coupled with a breakdown of the respective abundance and expression levels to individual AMR categories [*adj.p* < 0.05; Wilcoxon rank-sum test, * < 0.05, ** < 0.01, *** < 0.001]. **b** ARG abundance (top) and expression levels (bottom) of individual AMR categories significantly increased in COVID-19 patients compared to the control group [*adj.p* < 0.05; Wilcoxon rank-sum test, * < 0.05, ** < 0.01, *** < 0.001]

While co-infections or secondary infections in COVID-19 may exacerbate the disease, the presence of ARGs may limit treatment options. Since the overall abundance and expression of VFs was increased in COVID-19 individuals, we assessed the antimicrobial resistance profile of the microbial community in the COVID-19 and control groups. Specifically, using PathoFact, we characterized the prevalence and relative expression of ARGs (22 categories). While we did not find any significant differences in the overall gene abundances and the normalized expression levels of all ARGs contributing to the resistome, we observed a significant increase (*adj. p* < 0.05; Wilcoxon rank-sum test) in ARG alpha diversity (Supplementary Figure S3) between COVID-19 and the control groups (Fig. 4a). Importantly, when investigating individual AMR categories, we found that peptide resistance wash significantly higher in terms of gene abundance and also more highly expressed within the COVID-19 group (Fig. 4b; *adj. p* < 0.05; Wilcoxon rank-sum test). In addition, we observed that the expression of multi-drug resistance was enriched (*adj. p* < 0.05; Wilcoxon rank-sum test) in the COVID-19 group, while macrolides, lincosamides and streptogramins (MLS) and beta-lactam resistance both exhibited a higher gene abundance in the same group (*adj. p* < 0.05; Wilcoxon rank-sum test).

**Fig. 5:**
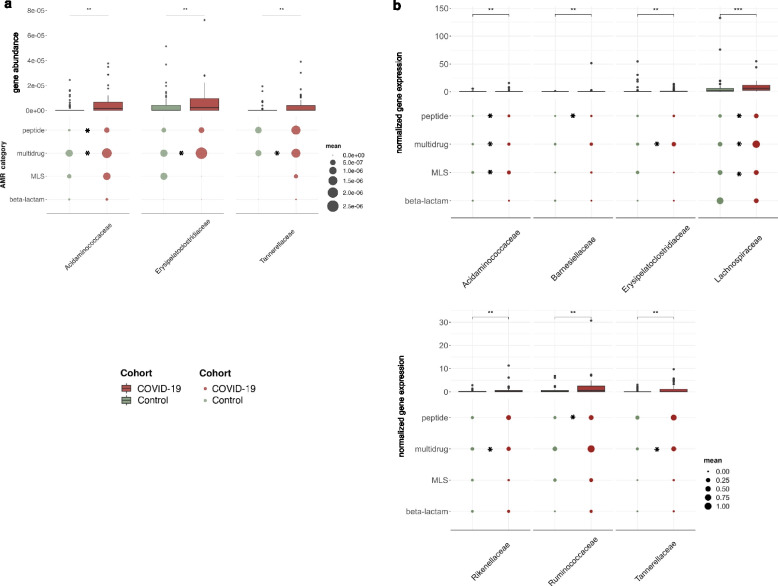
Association of AMR with the microbial community. Abundance (**a**) and expression (**b**) levels of ARGs and corresponding to AMR categories linked to MAGs. On top (boxplot) depicting the overall ARG abundance, below the average abundance of selected AMR categories per taxonomic family. The plot depicts taxonomic families in which overall a significant increase in abundance or expression of ARGs was observed [*adj.p* < 0.05; Wilcoxon rank-sum test, * < 0.05, ** < 0.01, *** < 0.001]

As described above, we leveraged the MAGs to correlate the differentially abundant and expressed ARGs to the microbial community. In line with our observations with the VFs, we found a significant increase (*adj. p* < 0.05; Wilcoxon rank-sum test) in ARGs encoded and expressed by the Acidaminococcaceae and Erysipelatoclostridiaceae in the COVID-19 group (Fig. 5a, b). Furthermore, an additional family, i.e., Tannerellaceae was also associated with increased abundance and expression of ARGs in the COVID-19 group (Fig. 5a, b). Specifically, in relation to the above-reported AMR categories, we identified a significant increase in multi-drug resistance encoded and expressed by all three of these taxonomic families. In addition, the Acidaminococcaceae also were found to encode a significant increase in ARGs contributing to peptide resistance. Interestingly, we found that several other taxonomic families were also associated with increased ARG expression in the COVID-19 group (*adj. p* < 0.05; Wilcoxon rank-sum test), although their gene abundances did not demonstrate any significant differences (Fig. 5b). These included Barnesiellaceae, Lachnospiraceae, Ruminococcaceae, and Rikenellaceae.

To further validate our findings, especially those linking VFs and ARGs with taxa, we used bias correction-based analyses for microbial compositions (ANCOM-BC). For both abundances and relative expression, across VFs and ARGs, ANCOM-BC revealed similar taxonomic families were enriched in the COVID-19 group, as identified in our initial analyses using MaAsLin2 and subsequent non-parametric tests. Based on the ANCOM-BC analyses, the COVID-19 group had a higher log2 fold-change of Erysipelatoclostridiaceae, Acidaminococcaceae, and Erysipelotrichaceae in the VFs compared to the control group (Supplementary Figure S4a-b). Similarly, ARGs in the Acidaminococcaceae and Erysipelatoclostridiaceae families were both abundant and showed higher relative expression in the COVID-19 group (Supplementary Figure S4 c-d).

### Infective competence of the gut microbiome

**Fig. 6:**
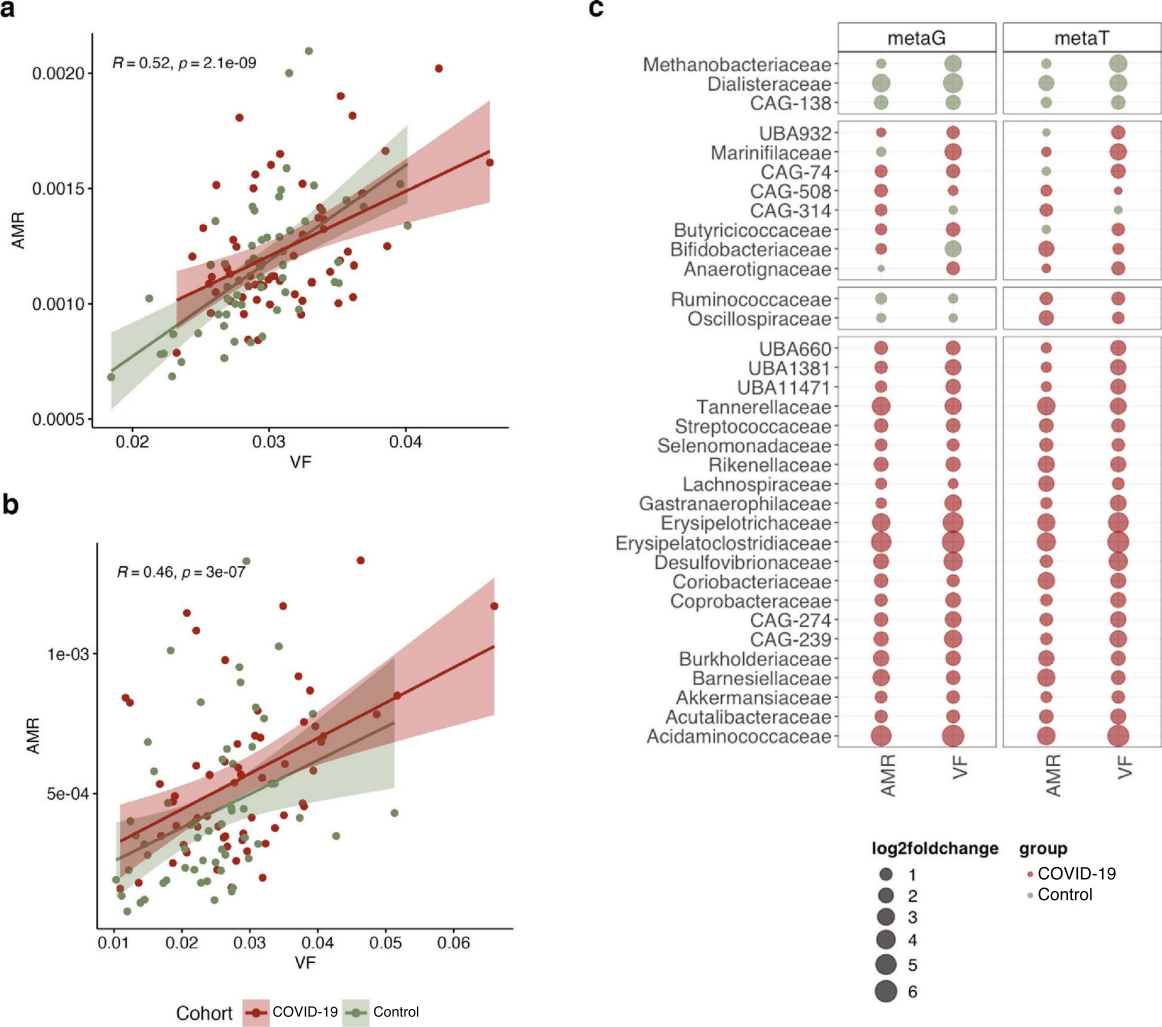
Assessing the *infective competence* of the gut microbiome. **a** Correlation of gene abundances of AMR and virulence factors [*R* = 0.52 and *p* < 0.01; Spearman’s correlation] in COVID-19 patients (red) and the control group (green). **b** Correlation of AMR and virulence factors gene expression levels [*R* = 0.46 and *p* < 0.01; Spearman’s correlation] in COVID-19 patients (red) and negative controls (green). **c** Bubble plot depicting the *infective competence* via the log2 fold change of AMR and virulence factors between COVID-19 patients (red) and control group (green)

Our analyses collectively indicated that both VFs and ARGs were enriched in abundance and expression in the COVID-19 group. Specifically, we found that the abundances of ARGs were correlated with those of the VFs (Fig. 6a, *R* = 0.52 and *p* < 0.01; Spearman’s correlation). Complementing this observation, we found that the expression profiles of ARGs and VFs also correlated with each other (Fig. 6b, *R* = 0.46 and *p* < 0.01; Spearman’s correlation) suggesting a higher propensity for infectious capacity. To further characterize the *infective competence* of the various taxa within the gut microbiome, we estimated the log2 fold-change of the abundance and expression of VFs and ARGs across taxonomic families found in the COVID-19 group and the control group. We found that ~ 62% (21/34) of the families had a higher *infective competence* and were enriched in abundance and expression within the COVID-19 group, whereas only ~ 9% (3/34) of the families showed increased *infective competence* in the control group (Fig. 6c). In particular, these analyses highlighted the Acidaminococcaceae and Erysipelatoclostridiaceae families*,* in line with our earlier observations, suggesting a higher *infective competence*, where the abundances and expression levels of the VFs and ARGs were significantly higher in COVID-19 compared to the control group (*p* < 0.05; two-way ANOVA). In the control group, Dialisteraceae, which was also observed earlier, showed increased *infective competence* (Fig. 6c). Collectively, our data suggests that the *infective competence* of taxa found in the COVID-19 group is increased compared to controls.

## Discussion

COVID-19 has become a common condition for which the manifold effects however remain a challenge [26]. Since the onset of the pandemic, the presentation of gastrointestinal symptoms has indicated the involvement of the gastrointestinal tract in COVID-19 [7]. As we uncover and understand the potential effects of COVID-19 in humans, it is important to also elucidate the concomitant consequences of the disease on the gut microbiome. To this end, several studies have focused on the drastic shifts in the microbiome of COVID-19 patients with severe symptoms. These include changes in diversity including stark enrichments and/or loss of specific taxa [27]. Several Studies have focused on differences in the gut microbiome between patients with severe COVID-19 and controls [7, 28]. Though these findings are essential, the effect on the larger population, wherein the infection is asymptomatic-to-moderate, is not readily represented. To address this particular gap in knowledge, we focused on the effect of COVID-19 in cases with asymptomatic-to-moderate symptoms in comparison to controls. Interestingly, we found that the diversity and overall shifts in community composition, highlighted in previous reports between severe and control patients [29], did not manifest themselves when comparing asymptomatic-to-moderate cases to the control group of individuals. However, this was associated with an increased abundances of specific taxa such as *Prevotella* spp., AM10 and CAG145 (Firmicutes phylum), *Roseburia* spp. and a *Turicibacter* spp. in the COVID-19 group. This is in contrast to existing reports [27, 30], suggesting the loss of beneficial taxa such as *Faecalibacterium*, *Bifidobacterium* and *Roseburia* in the context of COVID-19. Since our study did not include patients with severe COVID-19 or those that were hospitalized, it is likely that the lower disease severity does not lead to significant changes in the abundance of beneficial commensals. Along similar lines, major differences in the virome profile of the COVID-19 group in our study were not observed when compared to the control group. Nevertheless, and importantly, we found that genes associated with rotavirus C were increasingly expressed in the COVID-19 group, despite no differences in overall abundance of this virus between the patient groups. Rotavirus is a known enteric pathogen causing gastroenteritis in the pediatric population; however, their capacity to cause infections in adults is underappreciated and poorly characterized due to only mild symptoms including nausea, headaches, and diarrhea [31]. Importantly, at the time of writing only one report by Wang et al. [32], indicated the possibility of increased rotavirus A-mediated enteric infections in COVID-19 patients. These findings are intriguing given the propensity for COVID-19 patients to suffer from enteric symptoms [3], including nausea [33] and diarrhea [34]. Whether the rotavirus, especially in adults, is associated with COVID-19 gastrointestinal symptoms, or the enteric effects exacerbate the expression of rotavirus C-associated genes is still unknown and will have to be investigated in dedicated follow-up studies.

In line with the above observations, early in the pandemic, the role of COVID-19 in enhancing co-infections was documented extensively [14, 35]. This is not only limited to mucormycosis [36] which was amplified in certain parts of the world, but also bacterial and viral co-infections that were reported in severe COVID-19 patients [32]. Despite these observations and case studies, the effect of COVID-19 on the *infective competence* of the existing and endogenous microbiota has never been characterized. Our findings, therefore, bridge an important and broad chasm in knowledge, suggesting that COVID-19-mediated shifts may lead to higher microbiome-linked burden with potentially manifold effects. Importantly, we not only found an increased abundance in VFs in the COVID-19 group, but also a concomitant increase in expression of genes associated with virulence. Although it is plausible that a positive correlation between VFs and ARGs exists in de facto pathogens, such infection-linked shifts have not been reported beforehand. Furthermore, this phenomenon has not been reported in commensal organisms. Thereby, *infective competence* may be used to monitor and understand potential future infections in the context of COVID-19-mediated effects. Simultaneously, we found that these VFs were associated with taxa from families such as Acidaminococcaceae, Erysipelatoclostridiaceae, and Erysipelotrichaceae. Though Acidaminococcus was recently reported to be associated with a disease-related group in a large-scale meta-analysis [37], the exact role of Acidaminococcaceae in virulence is undocumented. Members of the Erysipelatoclostridiaceae family are typically seen as typical members of the microbiome; however, in specific cases species such as *Erysipelatoclostrium ramosum* have been associated with systemic infection and systemic inflammatory response syndrome [38], while Erysipelotrichaceae have been positively correlated with colorectal cancer [39]. Our observations, especially the increased expression of VF genes associated with these taxa, may pave the way in future explorations to serve as indicators of diseases. Importantly, it is still unclear whether the enriched *infective competence* is a COVID-19-specific hallmark or one found in all infections. For example, it has previously been hypothesized that selection of pathobionts result from inflammatory responses and/or a dysregulation of the tolerant immune system [40]. Future studies will need to address the extent to which various underlying factors such as a dysbiotic microbiome and an impaired immune system affect the *infective competence* of the gut microbiome. Furthermore, with larger cohorts which would include severe cases of COVID-19, supervised learning analyses may be employed to predict disease status based on the *infective competence* of individuals’ microbiome. Further work may also involve the heterologous expression of VF and AMR genes to assess predicted versus realized *infective competence*.

Another important aspect of COVID-19, in particular early on in the pandemic, was the overuse and misuse of antibiotics for treating SARS-CoV-2 [41] which was also associated with the potential increase of AMR [17]. Based on our findings and a recent report from the European Centre for Disease Prevention Control showing a North-to-South as well as a West-to-East AMR gradient in Europe during the COVID-19 pandemic [42], it is imperative to undertake future and detailed analyses accounting for socioeconomic and geographic factors contributing to AMR. In this context, Luxembourg may constitute an important reference population given its geographic location and its diverse demographic composition. Recent studies have reported on the higher incidence of AMR [43] and increased ARGs in COVID-19 patients [44]. However, these reports either refer to patients who were administered antibiotics [44] or include a meta-analyses observing datasets which were generated pre- and post-pandemic, specifically associated with travel [45], or limit characterization of antibiotic-mediated differences at a broad and low-resolution [46]. In contrast to these studies, antibiotic usage was a clear exclusion criterion in our study where individuals included were not administered any antibiotics 3 months prior to sampling. To our knowledge, our findings are the first report to systematically analyze the resistome of COVID-19 and control individuals and importantly to demonstrate that several of these ARGs are indeed expressed significantly higher in the COVID-19 group compared to the control group, regardless of antibiotic treatment. We observe that resistance genes include MLS, multi-drug and peptides, resistance classes where treatments of resistant bacteria are known to be inherently challenging with conventional antibiotics [47]. Strikingly, we found that the increased ARG expression in the COVID-19 group was further associated with the same taxa encoding and expressing VFs. This suggests that combinatorial effects of VFs and ARGs may exacerbate the *infective competence* of these taxa. This is further supported by our analysis identifying that taxa from the Acidaminococcaceae and Erysipelatoclostridiaceae families demonstrated a predicted higher *infective competence* in the COVID-19 group.

## Conclusions

Our findings suggest that it is imperative to elucidate all the implications of SARS-CoV-2 infection, especially its effect on the gut microbiome community and functions. Although other studies have involved the severe cases of COVID-19 [7, 48], none of these studies include both metagenomic and metatranscriptomic sequencing data. We found that the VFs and ARGs were indeed expressed in higher levels in the COVID-19 group compared to the controls. These key findings would have not been possible by only focussing on metagenomic data. Our collective findings, indicating the enriched abundance and expression of both VFs and ARGs, suggest that COVID-19 may yet have unknown effects that may come to light in the longer term including the shaping of the microbiome across the population. Moreover, we find that none of the commonly reported pathogens *(Salmonella*, *Shigella*, *Klebsiella* etc.) are enriched in the COVID-19 group in our study. In contrast, we find changes in *Prevotella* spp, AM10 and CAG145 (Firmicutes phylum), *Roseburia* spp and a *Turicibacter* spp. Therefore, it will be critically important to evaluate and further validate the effects of COVID-19 on the gut microbiome also in relation to infections by other viral and other pathogens. In particular, it remains unclear at this time, whether infections with other viruses, known to cause respiratory and gastrointestinal distress, e.g., Adenoviruses, respiratory syncytial virus (RSV), influenza viruses, norovirus, would lead to similar community and functional changes within the gut microbiome. Overall, it must be reiterated that pandemic preparedness coupled to the monitoring of VFs in tandem with antibiotic stewardship may be essential components for future strategies to mitigate the longer-term effects of COVID-19 and possibly other viral infections.

## Methods

### Cohort description and patient involvement

Between May and October 2020, stool samples were collected from 61 participants with COVID-19 confirmed by positive SARS-CoV-2 RT-qPCR (Supplementary Table S1) within the framework of the Predi-COVID study [49]. In order to be eligible to participate in the study, an individual must have been residing in Luxembourg and met all the following criteria: (1) signed informed consent form; (2) individuals ≥ 18 years old with confirmed SARS-CoV-2 infection as determined by PCR, performed by one of the certified laboratories in Luxembourg; and (3) hospitalized or at home. In addition to the criteria specific to the Predi-COVID study, samples were excluded if antibiotic treatment was reported. From the individuals, relevant clinical data was collected using a modified version of the International Severe Acute Respiratory and Emerging Infection Consortium (ISARIC) case report form. The participants to be included in the study were classified using an adapted version of the National Institute of Healthy symptom severity scheme [50]. Briefly, the classification of COVID-19 severity was based on the classification of the National Institute of Health (NIH) in the USA. Disease was classified as moderate if an individual had SpO2 ≥ 94%, and shortness of breath and/or evidence of lower respiratory disease. If SpO2 < 94%, a ratio of arterial partial pressure of oxygen to fraction of inspired oxygen (PaO2/FiO2) < 300 mmHg, a respiratory rate > 30 breaths/min, or lung infiltrates > 50%, disease was classified as severe. Subsequently, only asymptomatic to moderate symptoms were reported.

Along with the samples from the COVID-19 confirmed participants, stool samples were collected from a group of 57 individuals who tested negative for SARS-CoV-2 by RT-qPCR, who were participants of the CON-VINCE study, a population-based cohort study which recruited a representative sample of the Luxembourg population, to serve as age-matched controls. Participation in the control group was excluded if matching any of the following criteria: (1) infection of SARS-CoV-2 prior to the study; (2) presence of fever and respiratory distress/cough not attributable to other known chronic disease; (3) usage of antibiotics up to three months prior to enrolment or first SARS-CoV-2 infection. The study design is presented in Fig. 1. Demographic characteristics of the study groups are summarized in Table 1 while additional metadata are included in Supplementary Table S1. Patients were not involved in setting the research questions or the outcome measures of this study.

**Table 1:** Demographic characteristics of the study groups

	COVID-19 (*N* = 61)	Controls (*N* = 57)	*P* value
Age	43.85 ± 11.92	42.12 ± 3.32	0.297
Sex			
Female	22 (36.07%)	22 (38.6%)	0.776
Male	39 (63.93%)	35 (61.4%)	
COVID-19 severity			
Asymptomatic	4 (6.56%)	N/A	N/A
Mild	45 (73.77%)		
Moderate	12 (19.67%)		
Hospitalization status			
Hospitalized	1 (1.64%)	N/A	N/A
Not hospitalized	60 (98.36%)		
COVID-19 symptoms			
Fever	35 (57.38%)	N/A	N/A
Runny nose	11 (18.03%)		
Sore throat	22 (36.06%)		
Smell and/or taste loss	32 (52.46%)		
Fatigue	40 (65.57%)		
Headache	40 (65.57%)		
Cough	33 (54.1%)		
Shortness of breath	12 (19.67)		
Diarrhea	15 (24.59%)		
Abdominal pain	1 (1.64%)		
Chest pain	9 (14.75%)		
Ear pain	5 (8.2%)		
Joint pain	6 (9.84)		
Muscle pain	30 (49.18%)		
Vomiting/nausea/vertigo	7 (11.47%)		

*N* Number of participants, *N/A* Not applicable

### Sample collection and processing

Stool samples were collected at home by individuals in Fecal Collection Tubes (Zymo Research). Samples and data were collected at the Integrated BioBank of Luxembourg (IBBL). Around 1 g of stool was sampled, diluted in 9 ml DNA/RNA Shield according to the manufacturer’s instructions. Prior to DNA/RNA extraction, stool samples were thawed on ice and aliquoted as follows: 250 µl of sample was aliquoted for DNA extraction, to which 250 µl of lysis solution (ZymoBIOMICS DNA Miniprep Kit; Zymo Research) was added and the sample was subsequently kept frozen at − 80 °C until DNA extraction was performed. Furthermore, another 700 µl was aliquoted for RNA extraction using ZR BashingBead Lysis Tubes (Zymo Research) and the RNeasy Mini Kit (QIAGEN).

### DNA and RNA extractions

DNA was extracted using the ZymoBIOMICS DNA Miniprep Kit according to the manufacturer’s instructions with the following modifications: samples were inactivated for 7 min at 70 °C prior to homogenization by milling for 3 cycles (5 min of cooling on ice between cycles) for 60 s at 6 m/s in a FastPrep-24 5 G (MP Biomedicals). Prior to DNA purification, a Proteinase K incubation step was performed: 5 µl of 20 mg/ml Proteinase K (New England Biolabs GmbH) was added to each sample and incubated for 30 min at 40 °C. The extraction was performed following the manufacturer’s instructions and DNA was eluted in 50 µl DNase/RNase-Free Water (prewarmed to 60 °C). An RNase treatment was performed by adding 2.4 µl of 20 mg/ml Monarch RNase A (New England Biolabs GmbH) to each sample followed by incubation for 10 min at 56 °C. DNA was purified and concentrated using ZR-96 DNA Clean-Up Kit (Zymo Research) following the manufacturer’s instructions and DNA was eluted in 50 μl DNase/RNase-Free water (prewarmed to 60 °C). DNA was quantified using Qubit dsDNA BR assay kit (Invitrogen) and purity determined using Nanodrop 2000C (Thermo Scientific). Samples were frozen at − 80 °C until further use.

Samples for RNA extraction were inactivated for 7 min at 70 °C and 600 µl of cold RLT Buffer (containing 10 µl/ml 2-mercaptoethanol) was added to the samples prior to homogenization by milling for 3 cycles (5 min of cooling on ice between cycles) for 60 s at 6 m/s in a FastPrep-24 5 G (MP Biomedicals). Samples were centrifuged for 3 min at full speed and the supernatant was mixed with 1 volume of 70% Ethanol. Lysates were loaded onto a RNeasy Mini Spin Column and centrifuged at 8000 × *g* for 1 min. This last step was repeated until all supernatants had passed through the filters. Columns were washed according to the manufacturer’s instructions whereby 50 μl RNase-free water was added to the centre of the filter and incubated at room temperature for 1 min. RNA was eluted by centrifugation at 8000 × *g* for 1 min. RNA extracts were filled up to 87.5 μl with RNase-free water, 2.5 µl DNase I stock solution and 10 µl Buffer RDD (both RNase-Free DNase Set, QIAGEN) were added, mixed and incubated for 10 min at room temperature. RNA was purified and concentrated using RNA Clean & Concentrator-5 kit (Zymo Research) following the manufacturer’s instructions. RNA was eluted in 15 μl DNase/RNase-Free water. One microliter of obtained RNA was heat‐denatured for 2 min at 72 °C and quality-checked using Agilent RNA 6000 Nano kit (Agilent Technologies). RNA was quantified using Qubit RNA HS assay kit (Invitrogen). RNA extracts were frozen at − 80 °C for further use.

### Metagenomic and metatranscriptomic sequencing

DNA and RNA were extracted from all collected stool samples and sequenced for metagenomic and metatranscriptomic analysis, respectively. One hundred nanograms of DNA was used for metagenomic library preparation using Swift 2S turbo Flexible DNA library kit (cat. no. 45096). The genomic DNA was enzymatically fragmented for 10 min and DNA libraries were prepared without PCR amplification. The average insert size of libraries was 600 bp. Prepared libraries were quantified using Qubit (DNA HS kit, ThermoFischer) and quality-checked with a DNA HS kit on a Bioanalyzer 2100 (Agilient). Sequencing was performed at the LCSB sequencing platform (RRID: SCR_021931) on a NextSeq2000 instrument using 2 × 150 bp read lengths.

500 ng of RNA was rRNA depleted using the Illumina Ribo-Zero Plus rRNA Depletion kit (Illumina, 20,037,135). rRNA depleted samples were further processed using the TruSeq Stranded mRNA library preparation kit (Illumina, 20,020,594) which includes the fragmentation and priming steps. The fragmentation time was reduced to 3 min. Prepared libraries were quantified using Qubit (DNA HS kit, ThermoFischer) and quality checked with DNA HS kit on a Bioanalyzer 2100 (Agilient). Sequencing was performed at the LCSB sequencing platform (RRID: SCR_021931) on a NextSeq500 instrument using 2 × 150 bp read lengths. In total, this resulted in ~ 6 Gbp per sample for the metagenomics and ~ 21 Gbp per sample for the metatranscriptomics.

### Data processing, including genome reconstruction

The Integrated Meta-omic Pipeline (IMP; v3-commitID #b6f9da0e for preprocessing and #c04edbe for downstream assemblies) [25] was used for the processing and iterative co-assembly of metagenomic and metatranscriptomic reads. The workflow includes pre-processing, assembly, genome reconstruction, and functional and taxonomic annotation based on public and custom databases in a reproducible manner. For the data preprocessing, raw metagenomic reads were first trimmed to the maximal read length of 150 bases using Cutadapt (v3.4) [51]. The preprocessed metagenomic and raw metatranscriptomic reads were further processed using IMP: reads were trimmed using Trimmomatic (v.39) [52], reads mapping to the human genome (hg38 genome) or PhiX genome (gi|9,626,372|ref|NC_001422.1, Enterobacteria phage phiX174 sensu lato, complete genome) were removed using BWA (v. 0.7.9a) [53], and the metatranscriptomic reads were further filtered using SortMeRNA (v.4.2.0–238-g90cdf6c) [54]. In addition, alpha-diversity was calculated based on metagenomic reads using Nonpareil (v. 3.4.1) [55] as part of the IMP preprocessing step. Quality control was performed on the processed reads by running FastQC (v. 0.11.9) [56] and summarizing the reports using MultiQC (v. 1.10.1) [57]. In addition, Kraken2 (v. 2.1.2) [58] was used with a database containing only the human and PhiX genomes (https://ndownloader.figshare.com/files/24658262, from 11.09.2020, provided by Mike Lee) to confirm the successful removal of these contaminants from the processed sequencing data. The tool bbmap (v. 38.90) [59] was used on the preprocessed FASTQ files to extract reads mapping to SARS-CoV-2 reference genomes (same genomes as provided by fastv). Pairwise sample (dis)similarity was calculated using Mash (v. 2.3) [60].

*De novo* co-assembly of the processed metagenomic and metatranscriptomic reads was performed by running Megahit (v2.0) [61] included in IMP, followed by gene calling using an in-house modified Prokka version also allowing for incomplete ORFs [62]. Concurrently, MetaBAT2 [63] and MaxBin2 [64] together with an in-house binning methodology, binny [65], were used to reconstruct metagenome-assembled genomes (MAGs). Subsequently, we obtained a non-redundant set of MAGs using DAS Tool (v1.1.4) [66] with a score threshold of 0.7 for downstream analyses, and those with a minimum completion of 90% and less than 5% contamination as assessed by CheckM (v1.1.3) [67]. Taxonomy was assigned to the MAGs using gtdbtk (v1.7.0) [68]. Finally, MetaQUAST (v. 5.0.2) [69] was run on the created contig FASTA files to compute assembly statistics such as the number and maximal length of contigs, total assembly length, and the N50 and L50 values.

### Virome analyses

The co-assemblies built using metagenomic and metatranscriptomic data were used for the subsequent identification of viruses and to determine their functional activity. Briefly, the co-assembly was first processed through VIBRANT [70] and CheckV [71]. The CheckV assessment was repeated and any viral contigs with less than 70% completion were removed from further analyses. Subsequently, the complete viral contigs and those passing the 70% completion filter were merged and their respective taxonomies were determined using the IMGVR3 database [72]. To detect other viruses and confirm the status of SARS-CoV-2 infection in the processed reads, we also used fastv (v. 0.8.1, data for SARS-CoV-2 and for other viruses was downloaded on September 11th, 2021) [73]. Taxonomic consensus between the IMGVR3 and the fastv databases were determined to obtain overlapping, robust classification, and subsequently were used for the downstream analyses, where differentially abundant genes were further assessed for differential relative gene expression. 

### Prediction of microbial composition, virulence factors, and antimicrobial resistance

Profiling of the microbial community was performed on the processed reads using MetaPhlAn3.1 (v3.1.0, database “mpa_v31_CHOCOPhlAn_201901”) [74]. Simultaneously, profiling of antibiotic resistance factors was done using RGI (v5.2.0, CARD data v3.1.4, prevalence, resistomes and variants data v3.0.9) [75]. To obtain additional in-depth details of ARGs, in addition to the detection of VFs and mobile genetic elements (MGEs), PathoFact (v1.0; modified branch allowing the input of ORFs, #6fa64961) was run [24]. PathoFact is a pipeline for the prediction of ARGs and VFs, and their localization to MGEs, in metagenomic data. PathoFact was run on the contigs assembled by IMP together with their predicted protein sequences (ORFs) for each sample separately. PathoFact uses DeepARG [76] and RGI [75] for the prediction of ARGs, DeepVirFinder [77] and VirSorter [78] for the prediction of phages and PlasFlow [79] for the prediction of plasmids. Additionally, PathoFact uses its own developed tool, a combination of a HMM database (built on the VFDB [80]) and a random forest model, for the prediction of VFs. To run PathoFact, the input protein sequences were first processed to remove any trailing stop codon symbols (“*”) and to remove any sequence having an internal stop codon symbol as this is required for the tool RGI for ARG detection. For analyses of the predictions, FeatureCounts (v1.6.4) (Liao et al. 2014) was used to extract the number of reads per functional category. Thereafter, the relative abundance of genes and general expression levels was calculated using the Rnum_Gi method described by Hu et al. (Hu et al. 2013) which normalizes for both gene length and library size. Subsequently, metatranscriptomic expression levels were further normalized using the respective gene abundances from the metagenomic data (normalized gene expression = gene expression/ gene abundance).

### Statistical testing and data analysis

Statistical analyses of the taxonomic and functional data, as well as further visualizations, were performed using version 4.1.1 of the R statistical software package [81]. The R package MaAsLin2 [82] was used to determine associations between the cohort data and microbial features (e.g., functional and taxonomic profiles). Furthermore, MaAsLin2 identified significant differences were further validated by Wilcoxon rank-sum tests with adjustments using the ‘Benjamini-Hochberg’ method for multiple testing, specifically the ‘p.adjust’ function from the *stats* R package was used. To additionally, validate our findings with respect to the VF and ARGs, we used ANCOM-BC [83]. The *tidyverse, microbiomeViz*, *tidytree*, and *ggtree* packages were used to visualize the microbiome data, including using cladogram visualizations. The *tidyverse* package, including *ggplot2*, was used to generate all violin plots, box plots, and bubble plots. Finally, the *hmisc* and *corrplot* packages were used for all correlation plots.

## Supplementary Information

**Additional file 1:** **Supplementary Figure S1.** Dissimilarity of COVID-19 and control groups’ microbiome profiles. Alpha- (Shannon) (**a**) and beta-diversity (**b**) metrics of the gut microbiome compositions of the COVID-19 and control groups.

**Additional file 2:** **Supplementary Figure S2.** VF diversity between the COVID-19 and control groups. Alpha- (**a**) and beta-diversity (**b**) metrics of the VF abundances are depicted along with the VF relative expression alpha- (**c**) and beta-diversity (**d**) between the COVID-19 and control groups.

**Additional file 3:** **Supplementary Figure S3.** AMR diversity between the COVID-19 and control groups. Alpha- (**a**) and beta-diversity (**b**) metrics of the AMR abundances. AMR relative expression alpha- (**c**) and beta-diversity (**d**) between the COVID-19 and control groups.

**Additional file 4: Supplementary Figure S4.** Analysis of VF and ARG compositionality using ANCOM-BC. Log2 fold change (LFC) of (**a**) abundance and (**b**) normalised gene expression of VFs per taxonomic family between the COVID-19 and control groups. ARG abundance (**c**) and normalised gene expression (**d**) differences between the COVID-19 and control groups.

**Additional file 5: Supplementary Table 1. **Cohort metadata and description.

**Additional file 6:** **Supplementary Table 2.** Identified and significantly enriched viral genes in the COVID-19 group compared to control individuals.

## Data Availability

All code used for metagenomic and metatranscriptomic data analysis can be found in the following repository: https://gitlab.lcsb.uni.lu/ESB/co-infectomics. The repository at https://gitlab.lcsb.uni.lu/laura.denies/co-infectomics_r_analysis provides the code for the subsequent analyses in R. Individual workflows (sequencing data processing, QC, profiling, assembly, and analysis) were implemented using Snakemake [84]. Preprocessed and filtered reads, i.e., those filtered against the human genome (*hg38*), were submitted to the Sequence Read Archive hosted by NCBI and can be found under the accession PRJNA890008. Associated metadata is included in the code repository listed above. Additional data including assemblies, taxonomic, virome, functional pathway profiles along with MultiQC reports, can be found on Zenodo under the following link: https://doi.org/10.5281/zenodo.7192682.
